# Supplementary material for: Efficient gene knockout and genetic interaction screening using the in4mer CRISPR/Cas12a multiplex knockout platform
Source: Nat Commun. 2024 Apr 27;15:3577. doi: 10.1038/s41467-024-47795-3 (PMC11055879; doi:10.1038/s41467-024-47795-3)
Supplement: Supplementary file 1 — Supplementary Information [file 41467_2024_47795_MOESM1_ESM.pdf]

Efficient gene knockout and genetic interaction screening using the in4mer CRISPR/Cas12a multiplex knockout platform

Anvar *et al.*

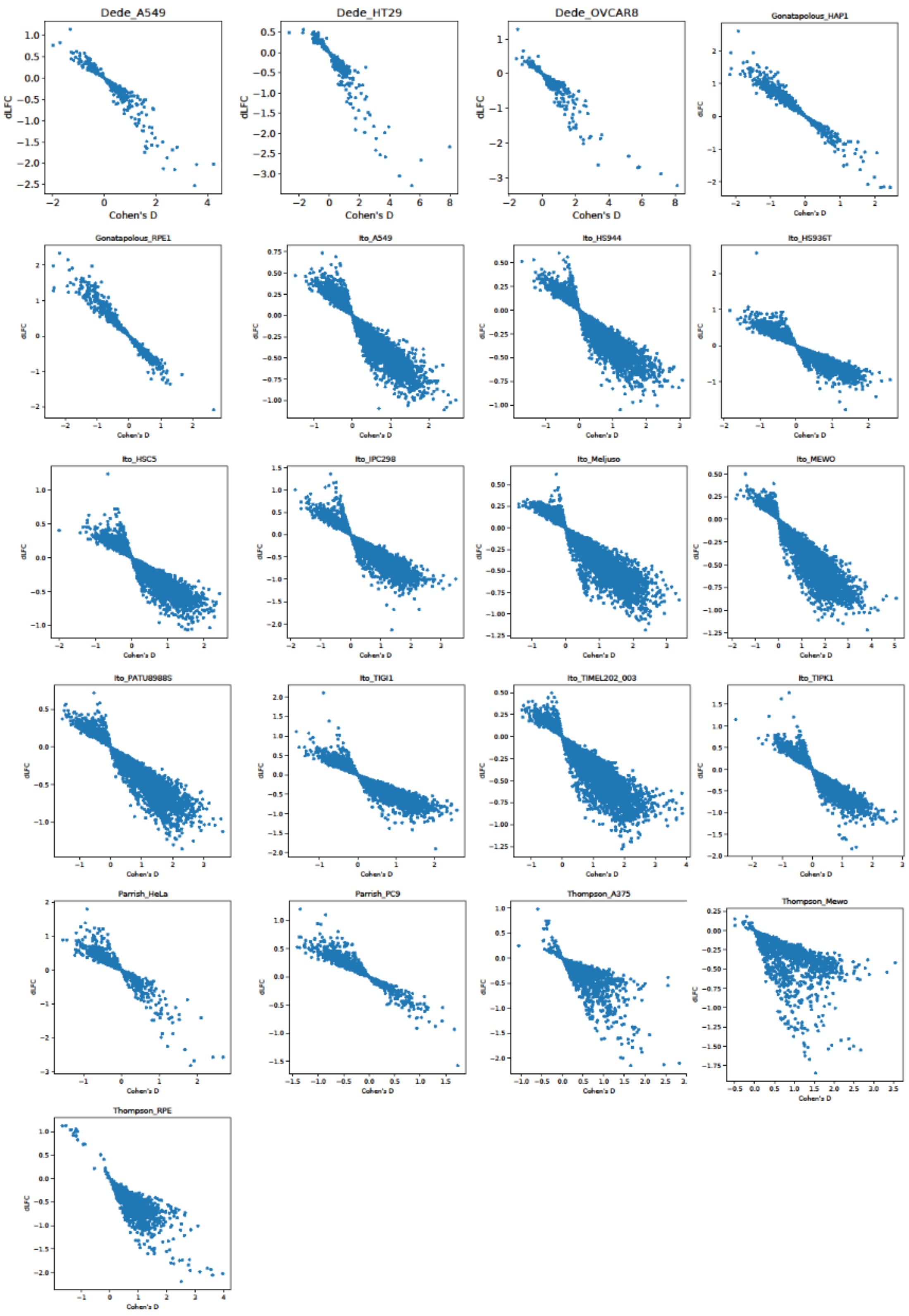

Supplementary Figure 1. **Cohen's d vs. dLFC for all cell lines in all five paralog screens.**

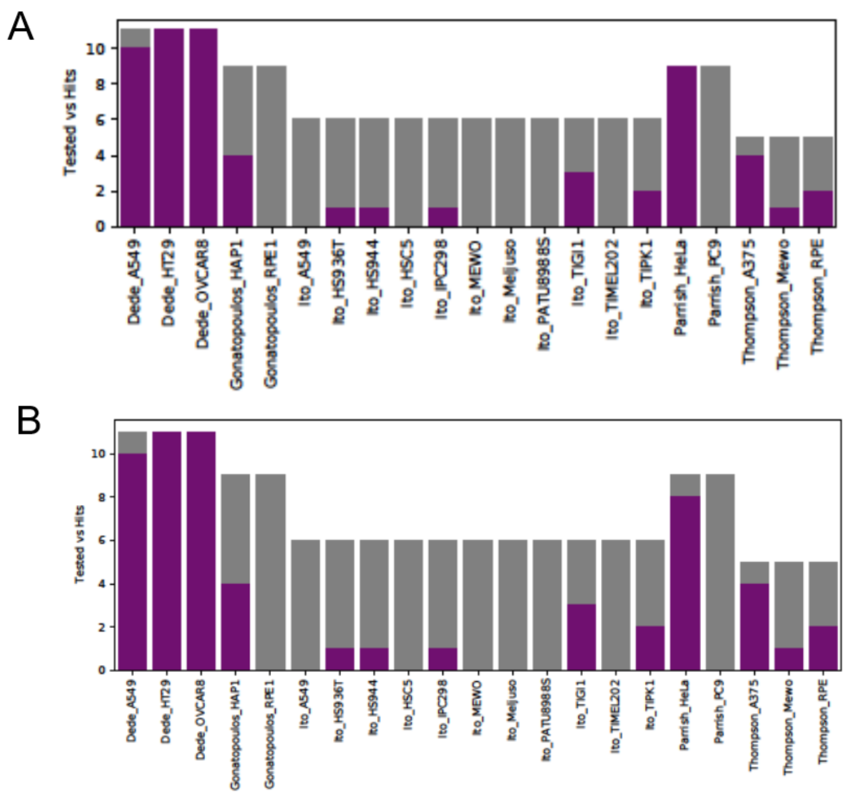

Supplementary Figure 2. Performance of paralog screens vs. candidate gold standards. (A) Across 21 cell line screens from 5 different platforms, number of candidate gold standards screened (gray) vs. hits (purple), measured by dLFC < -1. (B) Hits by Cohen's d > 0.8.

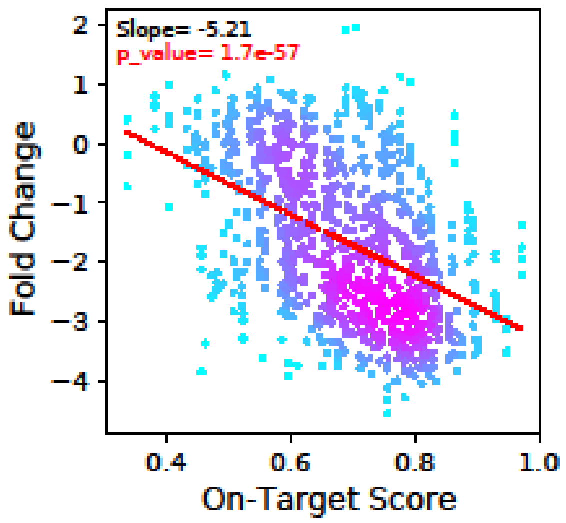

Supplementary Figure 3. Fold change vs. CRISPick on-target score for essential genes. Using CRISPick, more than 1000 positive control guides were selected targeting known essential genes. CRISPick on-target score (x-axis) is a good predictor of guide activity, as measured by fold change in a pooled library knockout screen (y-axis).

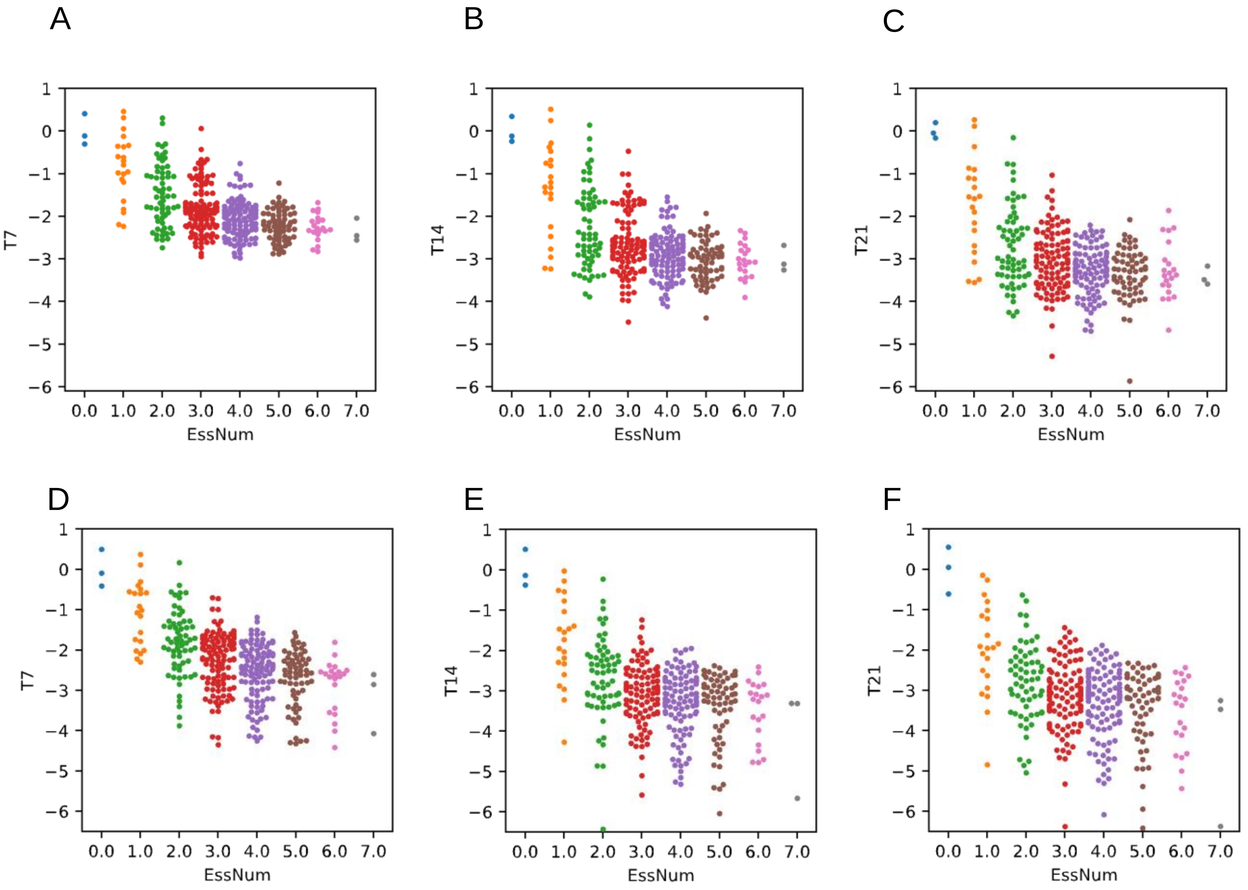

Supplementary Figure 4. Fold change of guide arrays vs. number of essential guides on the array in the 7mer library at different time points (n=384 arrays). Forward library, (A) Day 7, (B) Day 14, (C) Day 21 after puromycin selection. Reverse library, (D) Day 7, (E) Day 14, (F) Day 21 after puromycin selection.

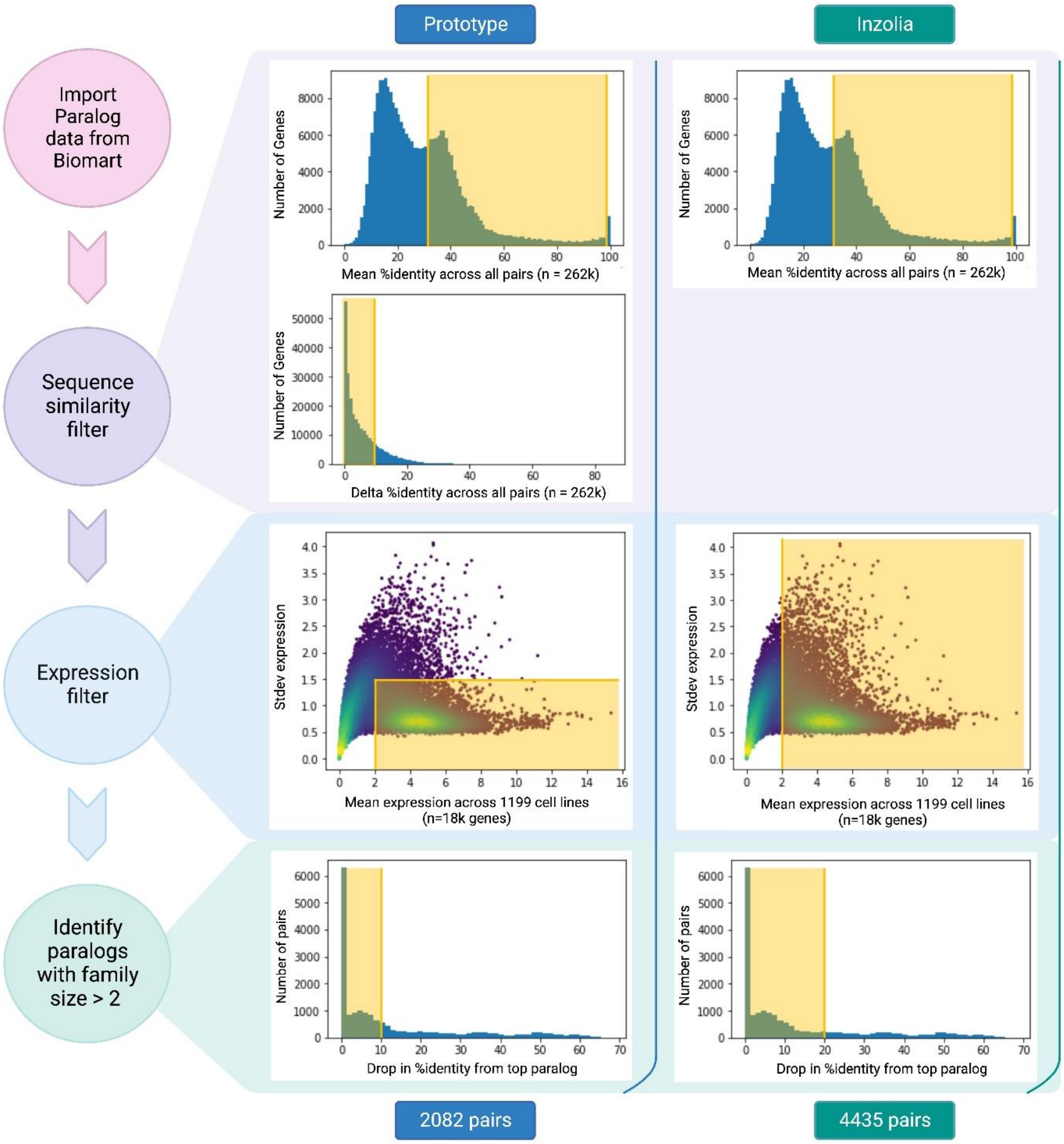

Supplementary Figure 5. Paralog selection pipeline, prototype, and Inzolia libraries.

A

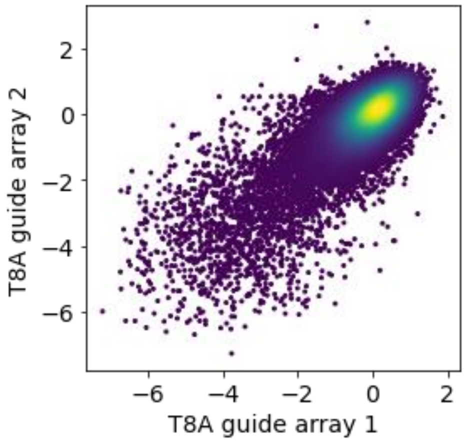

ρ=0.78

B

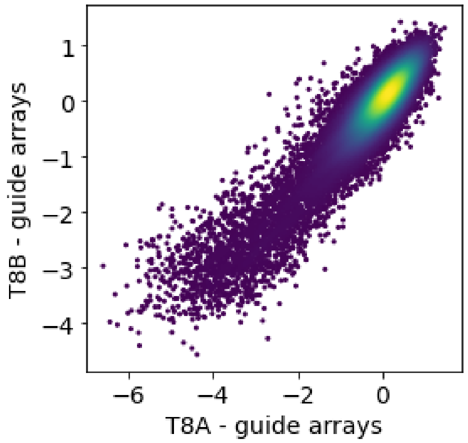

ρ=0.86

C

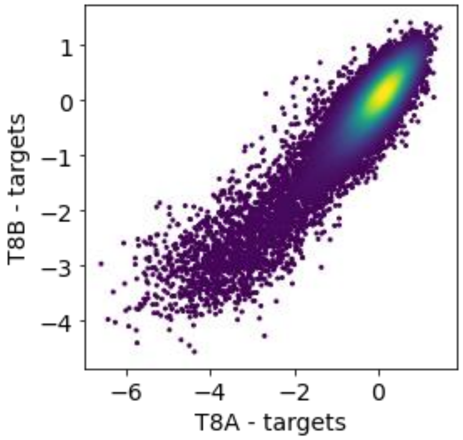

ρ=0.92

Supplementary Figure 6. Correlation within and between replicates at the array and gene levels. (A) log fold change (K-562 screen, T8) for guide arrays containing the same gRNA sequences in different order (n=21,783 common target sets). Pearson's ρ=0.78. (B) techincal/biological replicates (A,B) from one library transduction split into two replicates after puromycin selection. N=43,568 4plex guide arrays. Pearson's ρ=0.86. (C) mean of guide array fold changes targeting the same gene/gene set (n=21,784 targets), A vs B replicates. Pearson's ρ=0.92.

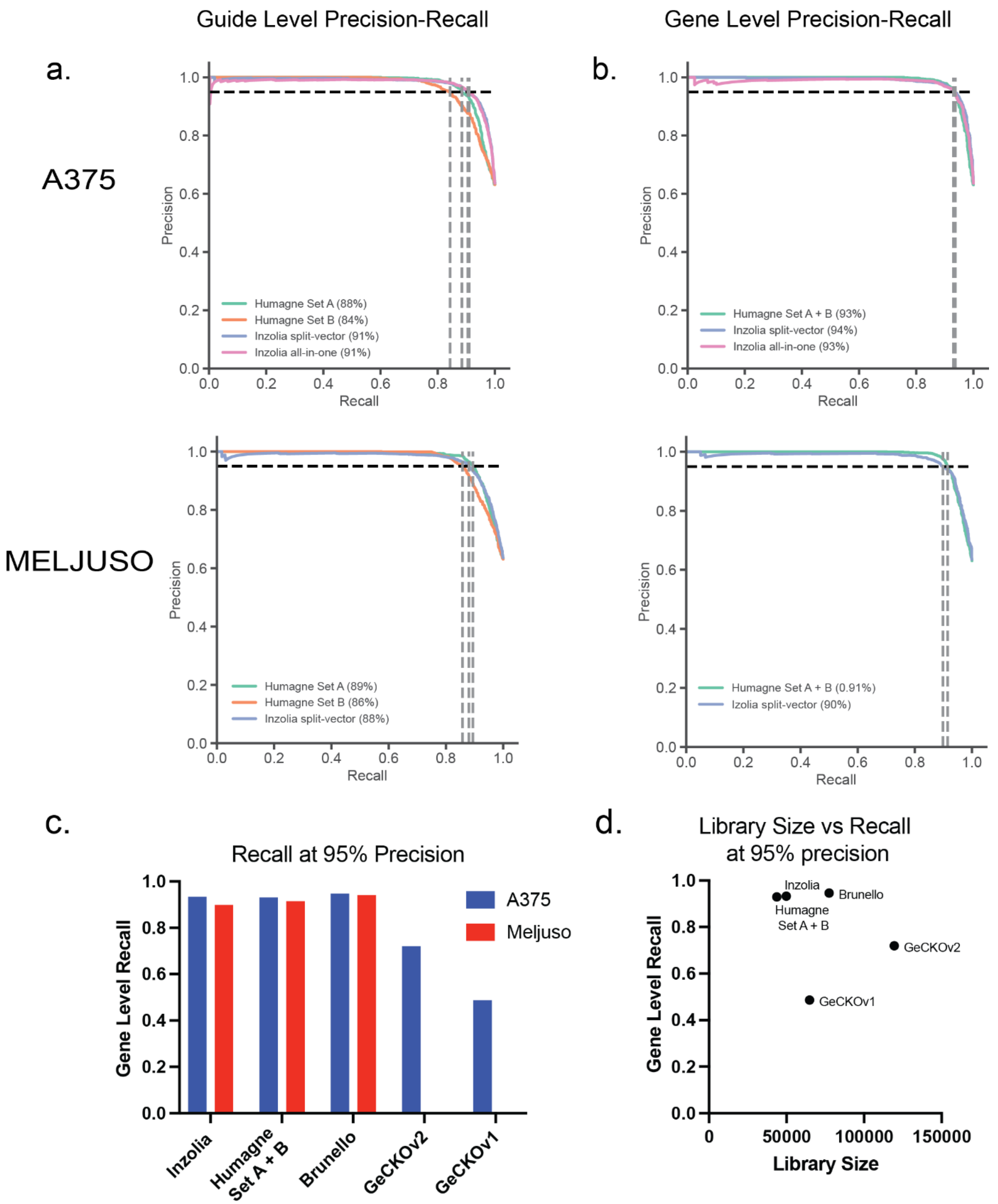

Supplementary Figure 7. Comparison of Inzolia and Humagne Sets A & B. (A) Guide level precision-recall curves for Humagne Set A, Humagne Set B, and Inzolia in A375 and MELJUSO cells. The recall at 95% precision is indicated for each curve in parentheses. (B) Gene level precision-recall curves for Humagne Set A + B, and Inzolia in A375 and MELJUSO cells. The recall at 95% precision is indicated for each curve in parentheses. (C) Gene level recall at 95% precision for selected genome-wide libraries in A375 and Meljuso. (D) Gene level recall at 95% precision compared to library size for selected genome-wide libraries.

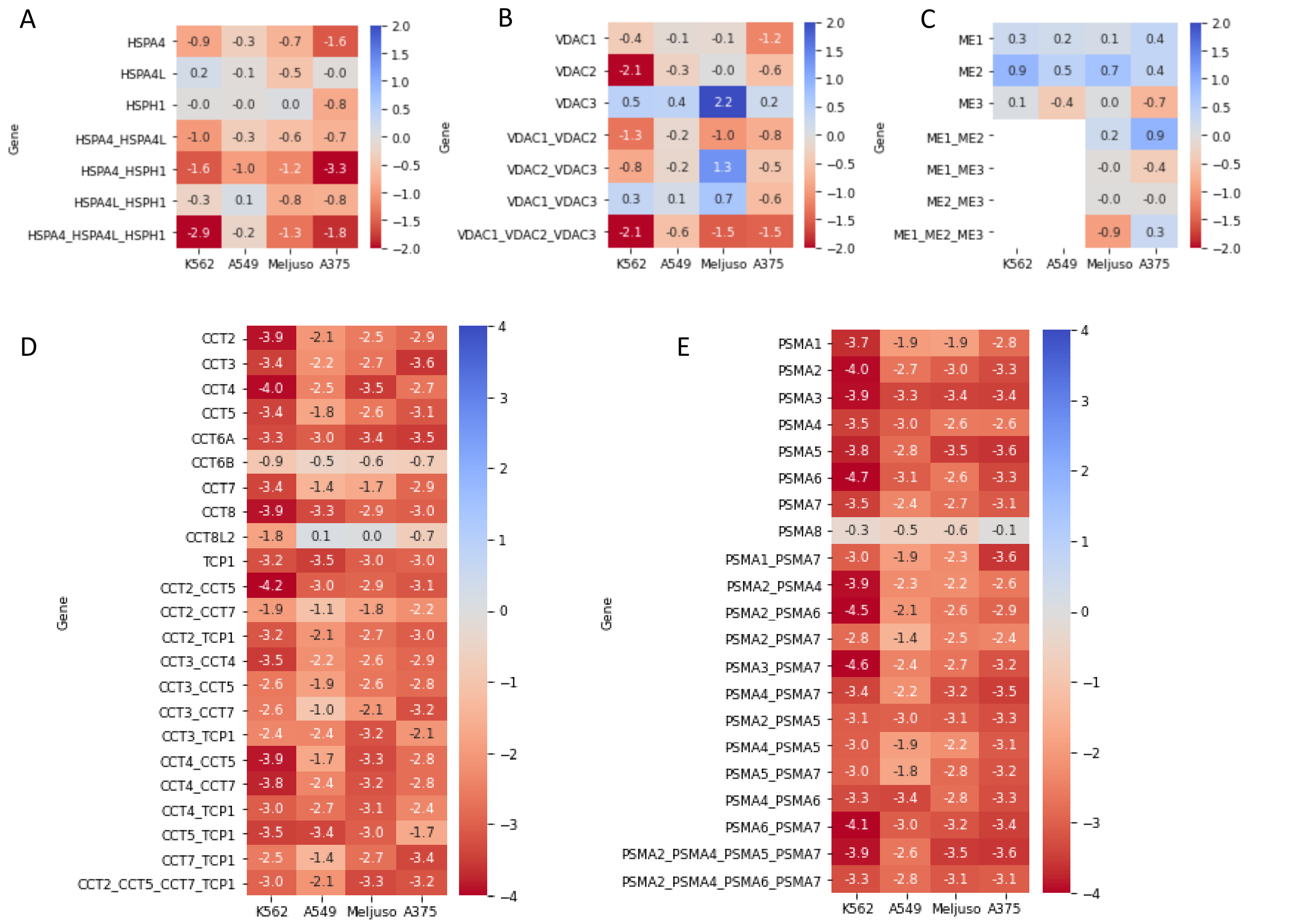

Supplementary Figure 8. Higher-order genetic interactions. (A) Candidate three-way synthetic lethal interactions among HSPA4 family members. (B) Candidate three-way interactions among VDAC1/2/3 family. (C) Candidate three-way interactions among ME1/2/3. (D-E) Masking/suppressor interactions among CCT complex and proteasome subunits. Single knockout induces severe loss of fitness and subsequent knockout of other subunits does not add to severity of phenotype.
