# Supplementary material for: Efficient gene knockout and genetic interaction screening using the in4mer CRISPR/Cas12a multiplex knockout platform
Source: Nat Commun. 2024 Apr 27;15:3577. doi: 10.1038/s41467-024-47795-3 (PMC11055879; doi:10.1038/s41467-024-47795-3)
Supplement: Supplementary file 3 — Description of Additional Supplementary Files [file 41467_2024_47795_MOESM3_ESM.pdf]

Description of Additional Supplementary Files

Supplementary Data 1. 388 Identified synthetic lethals, 26 synthetic lethals and gold standard set.

Supplementary Data 2. Raw read counts for 7mer screens forward.

Supplementary Data 3. Raw read counts for 7mer screens reverse.

Supplementary Data 4. Prototype library sequence.

Supplementary Data 5. Inzolia library sequence.

Supplementary Data 6. Raw read counts for both Prototype and Inzolia library screens.

Supplementary Data 7. Log fold change by gene for both Prototype and Inzolia library screens.

Supplementary Data 8. DrugZ score for MELJUSO screen with MEK inhibitor selumetinib.

Supplementary Data 9. GSEA result for MELJUSO screen with MEK inhibitor selumetinib.

Supplementary Data 10. Primers for library preparation and sequence.
