# Supplementary material for: Efficient gene knockout and genetic interaction screening using the in4mer CRISPR/Cas12a multiplex knockout platform
Source: Nat Commun. 2024 Apr 27;15:3577. doi: 10.1038/s41467-024-47795-3 (PMC11055879; doi:10.1038/s41467-024-47795-3)
Supplement: Supplementary file 14 — Reporting Summary [file 41467_2024_47795_MOESM14_ESM.pdf]

nature portfolio

Corresponding author(s): Traver Hart

Last updated by author(s): Apr 2, 2024

Reporting Summary

Nature Portfolio wishes to improve the reproducibility of the work that we publish. This form provides structure for consistency and transparency in reporting. For further information on Nature Portfolio policies, see our Editorial Policies and the Editorial Policy Checklist.

Statistics

For all statistical analyses, confirm that the following items are present in the figure legend, table legend, main text, or Methods section.

n/a	Confirmed	
☐	☒	The exact sample size (*n*) for each experimental group/condition, given as a discrete number and unit of measurement
☒	☐	A statement on whether measurements were taken from distinct samples or whether the same sample was measured repeatedly
☐	☒	The statistical test(s) used AND whether they are one- or two-sided<br>*Only common tests should be described solely by name; describe more complex techniques in the Methods section.*
☐	☒	A description of all covariates tested
☐	☒	A description of any assumptions or corrections, such as tests of normality and adjustment for multiple comparisons
☐	☒	A full description of the statistical parameters including central tendency (e.g. means) or other basic estimates (e.g. regression coefficient) AND variation (e.g. standard deviation) or associated estimates of uncertainty (e.g. confidence intervals)
☐	☒	For null hypothesis testing, the test statistic (e.g. *F*, *t*, *r*) with confidence intervals, effect sizes, degrees of freedom and *P* value noted<br>*Give P values as exact values whenever suitable.*
☒	☐	For Bayesian analysis, information on the choice of priors and Markov chain Monte Carlo settings
☒	☐	For hierarchical and complex designs, identification of the appropriate level for tests and full reporting of outcomes
☐	☒	Estimates of effect sizes (e.g. Cohen's *d*, Pearson's *r*), indicating how they were calculated

Our web collection on statistics for biologists contains articles on many of the points above.

Software and code

Policy information about availability of computer code

Data collection	External data in this study for meta-analysis in Figure 1 and Figure 6 are based on previous published data from: Parrish et al. 2021, Gonatopoulos-Pournatzis et al. 2020, Ito et al. 2021, Thompson et al. 2021 and Dede et al. 2020. The data from the meta-analysis is available at : 10.6084/m9.figshare.24243832
Data analysis	Meta-analysis of 5 dual-knockout CRISPR studies, Inzolia library design, In4mer data analysis were conducted in Python 3.8.10, using the packages Pandas 2.1.4, SciPy 1.11.4, NumPy 1.26.2, glob and statistics. Seaborn 0.13.0 and Matplotlib 2.8.2 were used to generate plots and upsetplot 0.9.0 package was used for upset plot generation.

For manuscripts utilizing custom algorithms or software that are central to the research but not yet described in published literature, software must be made available to editors and reviewers. We strongly encourage code deposition in a community repository (e.g. GitHub). See the Nature Portfolio guidelines for submitting code & software for further information.

Data

Policy information about availability of data

All manuscripts must include a data availability statement. This statement should provide the following information, where applicable:

- Accession codes, unique identifiers, or web links for publicly available datasets
- A description of any restrictions on data availability
- For clinical datasets or third party data, please ensure that the statement adheres to our policy

All data for this study is available on Figshare at doi://10.6084/m9.figshare.24243832
this DOI is reserved pending manuscript acceptance. Please use private link https://figshare.com/s/5ee5338a6d9a8dda95a9 during the peer review process.
Source Data are provided with this paper.

Research involving human participants, their data, or biological material

Policy information about studies with human participants or human data. See also policy information about sex, gender (identity/presentation), and sexual orientation and race, ethnicity and racism.

Reporting on sex and gender	N/A
Reporting on race, ethnicity, or other socially relevant groupings	N/A
Population characteristics	N/A
Recruitment	N/A
Ethics oversight	N/A

Note that full information on the approval of the study protocol must also be provided in the manuscript.

Field-specific reporting

Please select the one below that is the best fit for your research. If you are not sure, read the appropriate sections before making your selection.

☒ Life sciences ☐ Behavioural & social sciences ☐ Ecological, evolutionary & environmental sciences

For a reference copy of the document with all sections, see nature.com/documents/nr-reporting-summary-flat.pdf

Life sciences study design

All studies must disclose on these points even when the disclosure is negative.

Sample size	no sample sizes were determined. It is not clear what experiment is being asked about here. Sample size calculations do not appear to be a driver for any of these analyses.
Data exclusions	No data were excluded from the analyses.
Replication	Two replications of each screening.
Randomization	No external samples were collected or allocated to any experimental groups. All published data for the Fig 1 meta-analysis was collected and analyzed by the Hart group (NEA specifically). The question does not apply to our study.
Blinding	No investigators were blinded.

Reporting for specific materials, systems and methods

We require information from authors about some types of materials, experimental systems and methods used in many studies. Here, indicate whether each material, system or method listed is relevant to your study. If you are not sure if a list item applies to your research, read the appropriate section before selecting a response.

Materials & experimental systems

n/a	Involved in the study
☒	☐ Antibodies
☐	☒ Eukaryotic cell lines
☒	☐ Palaeontology and archaeology
☒	☐ Animals and other organisms
☒	☐ Clinical data
☒	☐ Dual use research of concern
☒	☐ Plants

Methods

n/a	Involved in the study
☒	☐ ChIP-seq
☒	☐ Flow cytometry
☒	☐ MRI-based neuroimaging

Eukaryotic cell lines

Policy information about cell lines and Sex and Gender in Research

Cell line source(s)	K562 and A549 cells were a gift from Tim Heffernan, A375 and MelJuso were obtained from the Cancer Cell Line Encyclopedia
Authentication	Cell line identities were confirmed by STR fingerprinting by M.D. Anderson Cancer Center's Cytogenetic and Cell Authentication Core
Mycoplasma contamination	Routinely tested by PlasmoTest Mycoplasma Detection Assay, InvivoGen. Cells are tested negative.
Commonly misidentified lines (See ICLAC register)	No commonly misidentified cell lines were used in the study
